# Morphology adjustable microlens array fabricated by single spatially modulated femtosecond pulse

**DOI:** 10.1515/nanoph-2021-0629

**Published:** 2022-01-04

**Authors:** Yang Liu, Xiaowei Li, Zhipeng Wang, Bin Qin, Shipeng Zhou, Ji Huang, Zhulin Yao

**Affiliations:** Laser Micro/Nano Fabrication Laboratory, School of Mechanical Engineering, Beijing Institute of Technology, Beijing 100081, China; Beijing Institute of Technology Chongqing Innovation Center, Chongqing 401120, PR China; National Institute of Metrology, Beijing 100029, China; AECC Shenyang Engine Research Institute, Shenyang 110066, China

**Keywords:** chemical etching, microlens, multiple-plane imaging, numerical-aperture, spatially modulated femtosecond laser pulse

## Abstract

Silica microlens arrays (MLAs) with multiple numerical-apertures (NAs) have high thermal and mechanical stability, and have potential application prospects in 3D display and rapid detection. However, it is still a challenge to rapidly fabricate silica MLAs with a larger range of NAs and how to obtain multiple NAs in the same aperture diameter. Here, a wet etching assisted spatially modulated femtosecond laser pulse fabricating technology is proposed. In this technology, Gaussian laser pulse is modulated in the axial direction to create a pulse with a large aspect ratio, which is used to modify the silica to obtain a longer modification distance than traditional technology. After that, a microlens with a larger NA can be obtained by etching, and the NA variable range can be up to 0.06–0.65, and even under the same aperture, the variable NA can range up to 0.45–0.65. In addition, a single focus is radially modulated into several focus with different axial lengths to achieve a single exposure fabricating of MLA with multiple NAs. In characterization of the image under a microscope, the multi-plane imaging characteristics of the MLA are revealed. The proposed technology offers great potential toward numerous applications, including microfluidic adaptive imaging and biomedical sensing.

## 1 Introduction

With the development of society and advancements in science and technology, the requirements for product integration have become more stringent. Microlens arrays (MLAs) are widely used in the aerospace industry, optics, microelectronics, communications, and other fields because of their small size, light weight, excellent optical performance, and high level of integration in applications such as bionic compound eye structures [1], [2], [3], 3D display [4, 5], virtual reality imaging [6], laser field homogenization [7], fiber coupling [8], and enhancement of light output [9]. Since different applications require different microlens morphologies, controlling the morphology of a microlens in MLA is a particularly crucial task. Many methods are used to fabricate microlens, such as photolithography [10], surface tension-assisted molding [11, 12], ink-jet printing [13], nanoimprinting [14], and dewetting [15]. However, fabricating hard and brittle materials with these methods is difficult because they are only intended for fabricating soft materials; in addition, soft materials do not work under extreme conditions, such as high-temperature and high-pressure environments.

Femtosecond laser is a widely used micro–nano tool for fabricating microlenses. Femtosecond laser can be used not only to fabricate polymer microlenses but also to fabricate hard and brittle material microlenses because of its high peak power [3, 16], [17], [18], [19], [20], femtosecond laser-assisted chemical etching (FLAE) can effectively improve the surface quality and fabricating efficiency of microlenses on hard and brittle materials [20], [21], [22], and can also adjust the NA of a microlens, whose adjustment range is up to 0.2–0.4 in the existing reports [23]. It is well known that a microlens with a small NA has a large depth of field, but will lose the imaging resolution; a microlens with a large NA has a high imaging resolution, but is limited by its small depth of field and cannot observe a larger range of objects. The MLA with multiple NAs solves this problem perfectly. However, how to fabricate MLAs with multiple NAs on hard and brittle materials with high efficiency and high quality is still a challenge.

Here, we propose a technology of fabricating MLA with multiple NAs in parallel that involves femtosecond laser spatial modulating and assistive wet etching. The incident one-spot single pulse Gaussian laser beam is shaped into multi-spot Bessel laser beams (BLBs) with different base angles (BAs) of phases by using a spatial light modulator (SLM). BLBs have a long focusing length and are often used to fabricate large aspect ratio microchannels and microholes [24, 25]. The focusing length of the BLBs with different BAs of phases is variable because focusing length is related to BA of Bessel phases. This property of BLBs can be used for the parallel fabrication of a material at different depths through adjustment of the BA in the Bessel phase. Then, wet etching can be used to form microlenses with different NAs. This technology is an effective parallel fabricating technology for microlenses, with a wide range of adjustable NAs (0.06–0.65), and even can realize microlenses with the same aperture diameter and different NAs in a certain BA range.

## 2 Experimental

### 2.1 Experimental setup and materials

**Figure 1:**
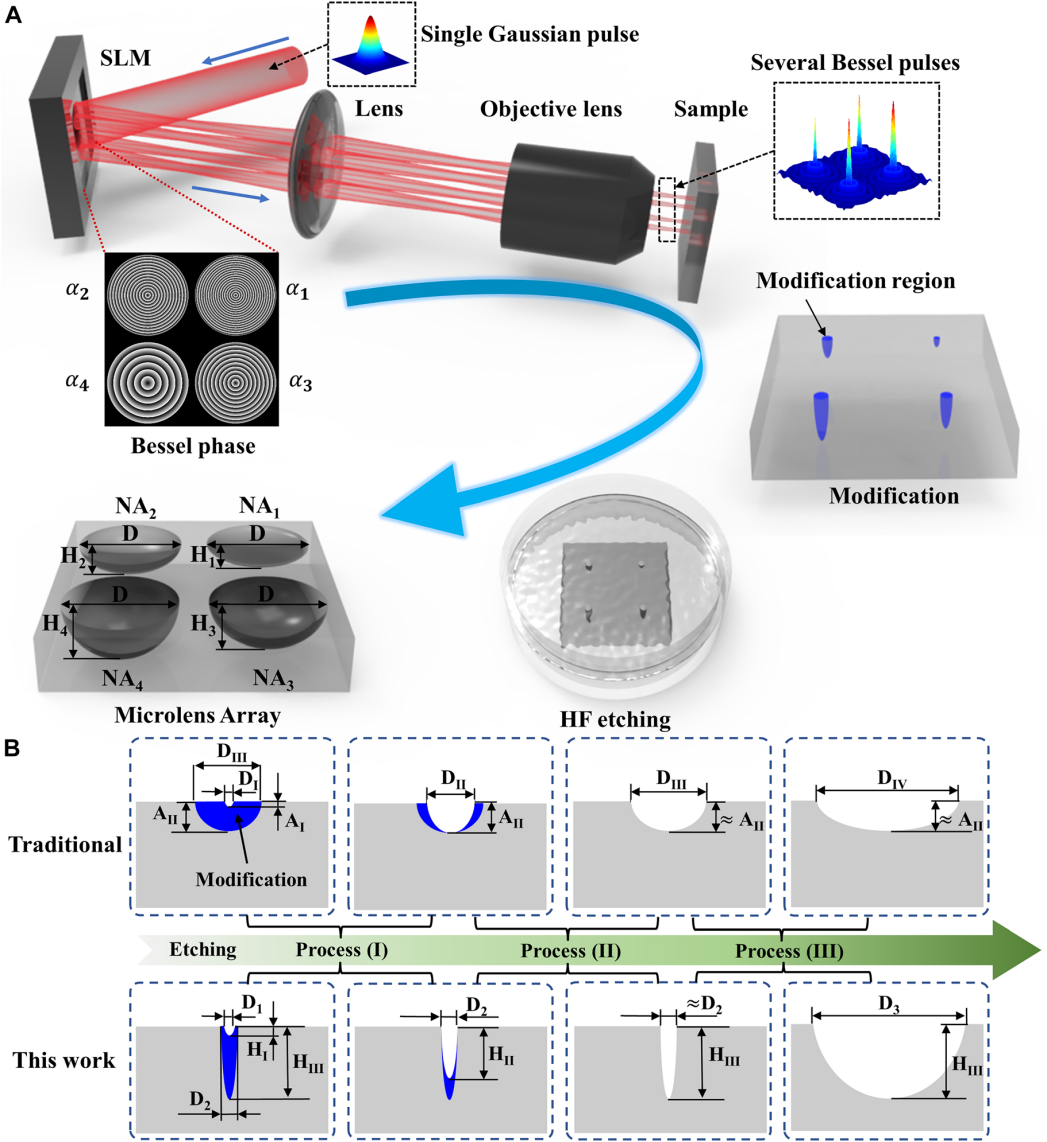
Schematic of the design and fabrication of an MLA with multiple NAs and analysis of etched procedure after modification. (A) Schematic of the design and fabrication of an MLA with multiple NAs. (B) Schematic comparison of traditional technology and proposed technology in the etching procedure. The blue region represents the modification region, the white default region represents the structure region. D represents the structure diameter. A and H represent the height of the structure fabricated by the traditional technology and the proposed technology, respectively.

The fabricating settings of an MLA with multiple NAs are shown in Figure 1A. A Bessel computer-generated hologram (CGH) was loaded into the SLM. A femtosecond laser was modulated by the SLM, which was loaded with the Bessel CGH consisting of four Bessel phases with different BAs to generate four BLBs with varying focusing lengths. The relationship between the length of a Bessel region and the BA is as follows:

(1) β=arcsin(n sin α)−α

(2) $$Z_{\text{max}}=\frac{w_{0}}{\text{tan }\beta }$$

where *β* is the exit angle, *α* is the BA, *n* is the refractive index of the axicon, which is 1.45, $Z_{\text{max}}$ is the length of the Bessel region before it passes through the telescopic system, and *w*_0_ is the beam radius at the waist of the incident Gaussian beam.

As Figure 1A depicts, the BLBs were demagnified to micro-BLBs through a telescope system consisting of a plano-convex lens and a microscope objective. The equations for the length of the Bessel region after the beam passes through the telescopic system are as follows:

(3) $$\Gamma =\frac{f_{1}}{f_{2}}$$

(4) $$Z_{\text{max}}^{\prime }=\frac{Z_{\text{max}}}{\Gamma^{2}}$$

where Γ is the demagnification ratio, *f*_1_ and *f*_2_ are the focal length of the plano-convex lens and the equivalent focal length of the microscope objective lens, which are 150 and 9 mm, respectively, and $Z_{\text{max}}^{\prime }$ is the length of the micro-Bessel region after the BLB passes through the telescopic system. Figure S1 presents illustration schematic of the parameters in Eqs (1)–(4).

Fused silica was selected as the material for fabricating. The four micro-BLBs were focused on the fused silica sample to form four modification regions of different volumes. The aperture diameters (D) and heights (H) of these volumes can be adjusted quantitatively by changing the BA in the Bessel phase. For example, the BA can be adjusted so that the microlenses etched in the array have the same D but different H ( ${\text{H}}_{1}\neq {\text{H}}_{2}\neq {\text{H}}_{3}\neq {\text{H}}_{4}$ ) to ensure they have different NAs ( ${\text{NA}}_{1}\neq {\text{NA}}_{2}\neq {\text{NA}}_{3}\neq {\text{NA}}_{4}$ ), as Figure 1A depicts.

### 2.2 Wet etching process

Different concentrations of hydrofluoric acid (HF) have different etching selectivity for the modified and unmodified regions of silica [26]. After comprehensive considering, the two requirements of the etching selectivity and the morphology of the microlens, we chose 20% HF as the etching agent after laser modification, and the temperature was room temperature. After etching, the sample was cleaned with water and ethanol for 10 min to ensure no HF residue remained and the sample surface was clean.

Schematic comparison of traditional technology and proposed technology in the etching procedure is shown in Figure 1B. We found that the etching procedure of the two technologies can be divided into three processes. Firstly, the etching procedure of traditional technology is described. Process I of the traditional technology: the modified region in the axial direction has been completely removed, the height of the structure increases from A_I_ to A_II_, and the diameter increases from D_I_ to D_II_. Usually in this process, the modified region in the radial direction has not been completely removed. Process II of the traditional technology: the modified region in the radial direction has been completely removed, and the diameter increases from D_II_ to D_III_. Since the etching rate of the modified region by hydrofluoric acid (HF) solution is much faster than that of the unmodified region, the increase in height can be ignored in this process. Process III of the traditional technology: the diameter of the structure continues to increase from D_III_ to D_IV_, but the height A_II_ almost unchanged because the bottom of the structure has the same etching rate as the sample surface. Therefore, the final microlens height depends on the initial modification depth. The proposed technology has a larger modification depth and a larger aspect ratio than that of the traditional technology. Moreover, Process I and the Process II of the two technologies are different. Process I of the proposed technology: the modified region in the radial direction has been completely removed, the diameter of the structure increases from D_1_ to D_2_, and the height increases from H_I_ to H_II_. Process II of the proposed technology: the modified region in the axial direction was completely removed, and the height increases from H_II_ to H_III_, but the increase in diameter can be ignored in this process. The detailed description of the etching procedure of the proposed technology is in Section 3.2 Analysis of etching process.

### 2.3 Sample characterization

The top view and morphology of the structures were characterized before etching by using scanning electron microscopy (SEM; XL30S-FEG, FEI, Inc.) and atomic force microscopy (AFM; Dimension Edge PSS, Bruker, Inc.), respectively. The 3D profile and height of the etched structures were characterized by using laser confocal scanning microscopy (LCSM; OLS4100, Olympus Inc.). The morphologic evolution of the structure with etching time was characterized by using an optical transmission microscopy (OTM; BX 51, Olympus Inc.). The basic internal structure of the fused silica was characterized by using a confocal Raman spectrometer (inVia Reflex, Renishaw Inc.).

## 3 Results and discussion

### 3.1 Morphology of microlens is controlled by single pulse

**Figure 2:**
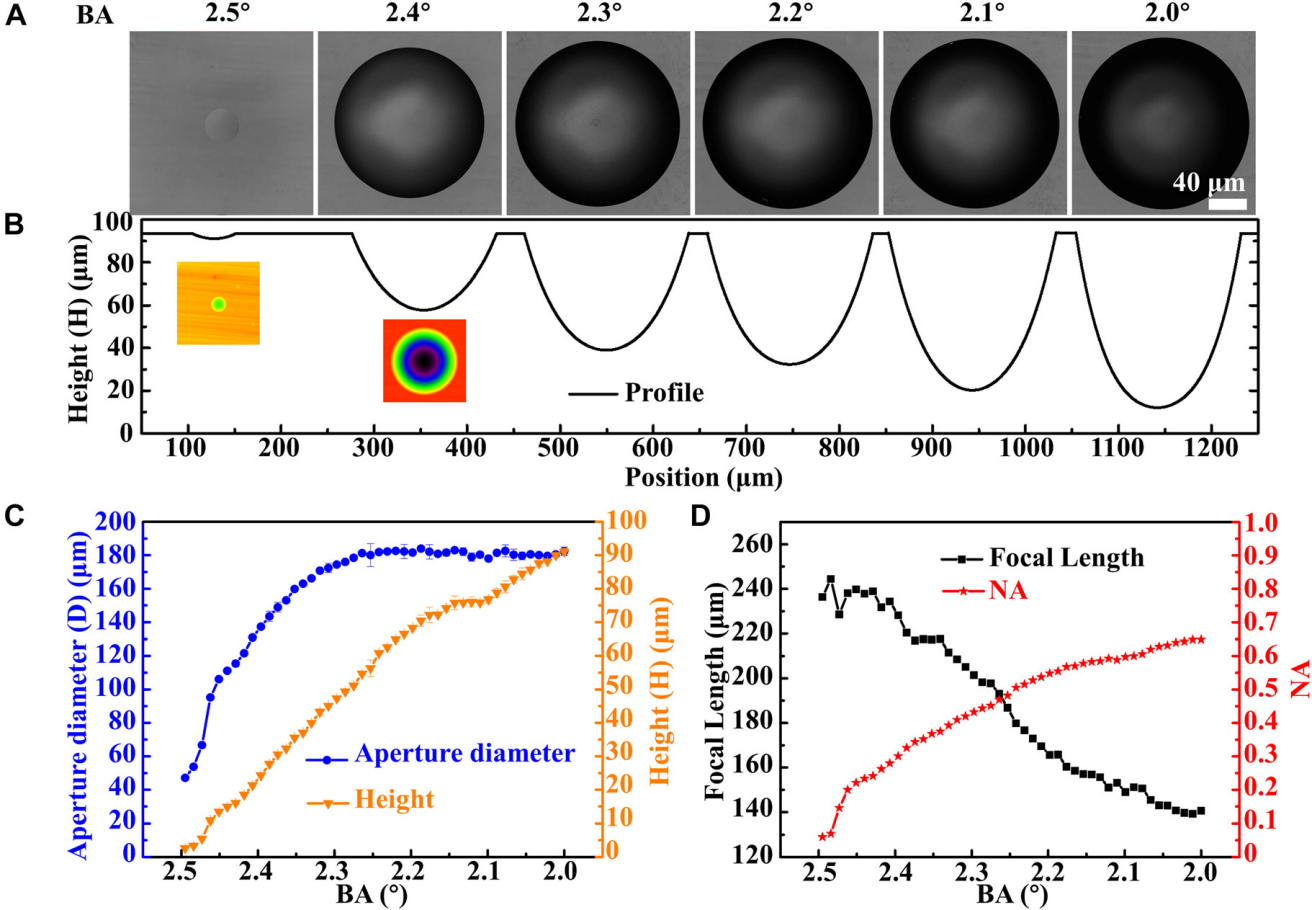
Microlenses defined by the BLBs with a variety of BAs. (A) LSCM top view of the microlenses after etching for 20 h. (B) Corresponding cross-section profiles of the microlenses in (A) and the pseudo-color images corresponding to 2.5° and 2.4° (inset images). (C) Aperture diameters and heights of the microlenses etched after exposure with different BAs. (D) Relationship among BAs, focal lengths (absolute value), and NAs of the microlenses.

First, we loaded a Bessel CGH with phase radius of 270 pixels (8 μm for each pixel) and a BA of 2.5° into the SLM. According to Eqs. (1) and (2), $Z_{\text{max}}$ was 110 mm, and according to Eq. (3), Γ was 16.67. Therefore, $Z_{\text{max}}^{\prime }$ , which is the length of the micro-Bessel region corresponding to the BA of 2.5°, was 395 μm according to Eq. (4). The micro-BLB formed after passing through the telescopic system just touched the sample surface, and the *z*-value of the translation stage was fixed for subsequent fabrication. It should be noted that if there was no special description, all operations in the following would no longer move the translation stage in *z* direction. In order to study the effect of BA on the microlens after etching. We replaced the BA in the Bessel phase CGH with 2.4°, 2.3°, 2.2°, 2.1°, 2.0°, respectively. According to Eqs. (1)–(4), the corresponding lengths of the micro-Bessel regions were 412 μm, 430 μm, 449 μm, 471 μm, and 494 μm, respectively. The difference between the length of these five micro-Bessel regions and 395 μm was the axial length of the focus inside the material, which were 17 μm, 35 μm, 54 μm, 76 μm and 99 μm, respectively. In previous reports, the maximum axial length of the focus was only 20 μm [23]. These BLBs were focused on the sample to form modified regions with different volumes, as shown in Figures S2 and S3. The LCSM images of the microlenses after etching 20 h are shown in Figure 2A, and the profiles of these microlenses are shown in Figure 2B. The surface roughness of the six microlenses were measured using LCSM, and the RMS were 4 nm, 9 nm, 10 nm, 5 nm, 7 nm and 5 nm. We reduced the step size in the change of the BA from 0.1° to 0.01° in the Bessel phase within the range of 2.5°–2.0°. Figure 2C presents the aperture diameters and heights of the corresponding microlenses after etching. We observed that as the BA in the Bessel phase decreased, the height of the microlens increased; and the aperture diameter increased at first but then remained almost unchanged. Figure 2D presents the focal length (absolute value) and NA of each microlens in Figure 2C according to Eqs. (S1)–(S3). The adjustable range of the microlenses’ NAs was 0.06–0.65.

**Figure 3:**
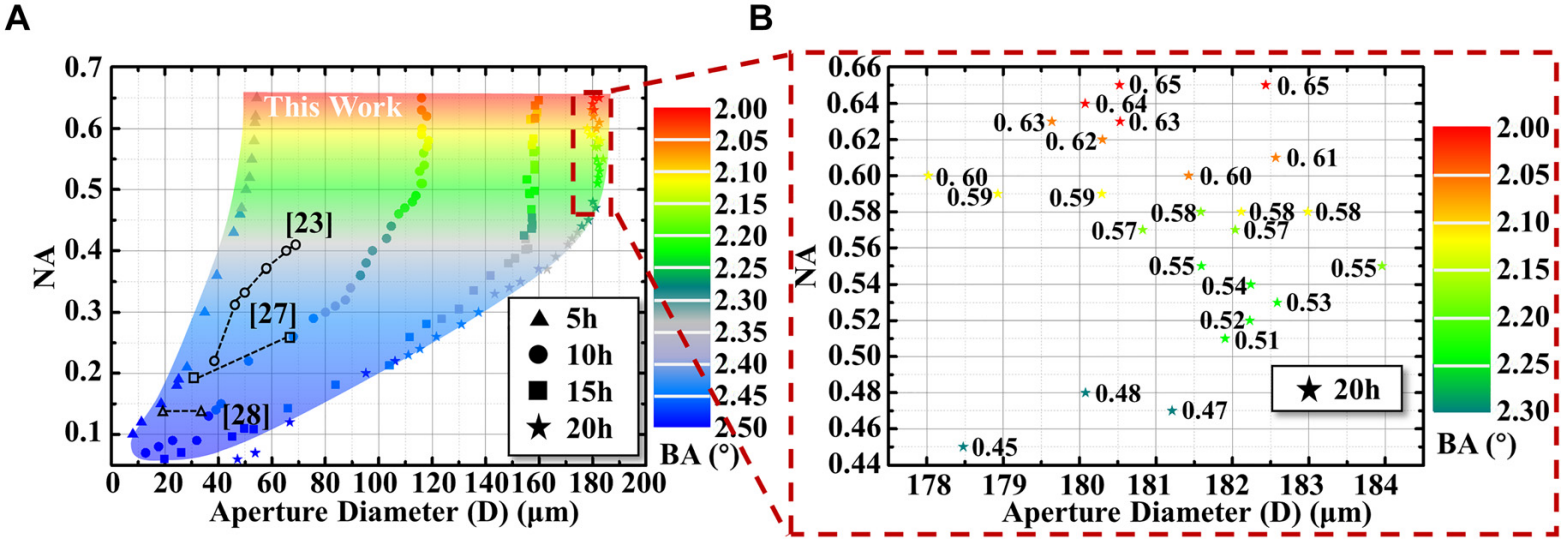
The aperture diameters and NAs of microlenses fabricated using the proposed technology at different etching time. (A) Relationship between aperture diameters and NAs of the microlenses fabricated by the BAs of the different BLBs and different etching time. And the aperture and NAs of the microlens in other reports. (B) Relationship between the aperture diameters from 178 to 184 μm in (A) and the NAs of the microlenses defined by the BAs of the different BLBs.

Figure 3A depicts the relationship between the aperture diameters and the NAs of the etched microlenses defined by the BAs of the different BLBs. For comparison, the relationship between the aperture diameters and NAs of the microlens fabricated by other reported FLAE technologies are also shown in Figure 3A [23, 27, 28], 0.06–0.65 is the maximum range to have ever been reported for fabricating microlenses using FLAE technology. In addition, we examined the maximum NA of microlens fabricated by femtosecond laser technology in other reports, the details were shown in Table S1, and the maximum value was only 0.5.

In order to facilitate the display of the four sets of data in Figure 3A, the etching time, the variation range of the BA, the variation range of microlenses aperture diameters after etching, and the corresponding NAs variation range are expressed in the form of four arrays: (5 h, 2.5°–2.33°, 8–54 μm, 0.1–0.65), (10 h, 2.5°–2.15°, 13–116 μm, 0.07–0.65), (15 h, 2.5°–2.11°, 20–160 μm, 0.06–0.65), (20 h, 2.5°–2.06°, 47–182 μm, 0.06–0.65). Although only the data corresponding to the four etching times are shown in Figure 3A, theoretically the color region in the figure can be filled by decreasing the step size of the etching time.

Generally, the NA of microlens fabricated by the FLAE technology is related to the aperture diameter [28], [29], [30], [31], [32]. However, the NAs of the microlenses fabricated through the proposed technology can be independent of the aperture diameters in a certain BA range. The maximum adjustable range of NA under the same aperture diameter is 0.45–0.65, and the difference is 0.2. For example, as Figure 3B depicts, with an etching time of 20 h and BAs of 2.3°–2.06°, the corresponding NAs are 0.45–0.65 in the aperture diameter range of 180 ± 2 μm. Moreover, a larger number of NAs with the same aperture diameter can be achieved by decreasing the increments between the BAs.

We also used the traditional technology to fabricate the microlens to compare with the proposed technology. The single pulse energies of the Gaussian laser pulses were 4–20 μJ. The LCSM images of the etched microlenses, and the relationship among energies, aperture diameters, and heights are shown in Figure S4. After calculation, the range of NAs adjustable by changing the energies of the Gaussian laser pulses was 0.12–0.28 (Figure S4). The LCSM images of the etched microlenses by varying the number of Gaussian laser pulses from 1 to 1000, and the relationship among the number of pulses, aperture diameters, and heights are shown in Figure S5. After calculation, the range of NAs adjustable by changing the numbers of pulses of the Gaussian lasers was 0.25–0.31. In addition, as the results (Figures S6 and S7) demonstrate, the NA ranges adjustable by changing either the energies or the pulse numbers of the BLBs were 0.23–0.44 and 0.23–0.28, respectively. Therefore, the NA of the microlens cannot be substantially changed by adjusting pulse energy or number of pulses, even if the Gaussian laser pulse was shaped into the Bessel laser pulse.

### 3.2 Analysis of etching process

**Figure 4:**
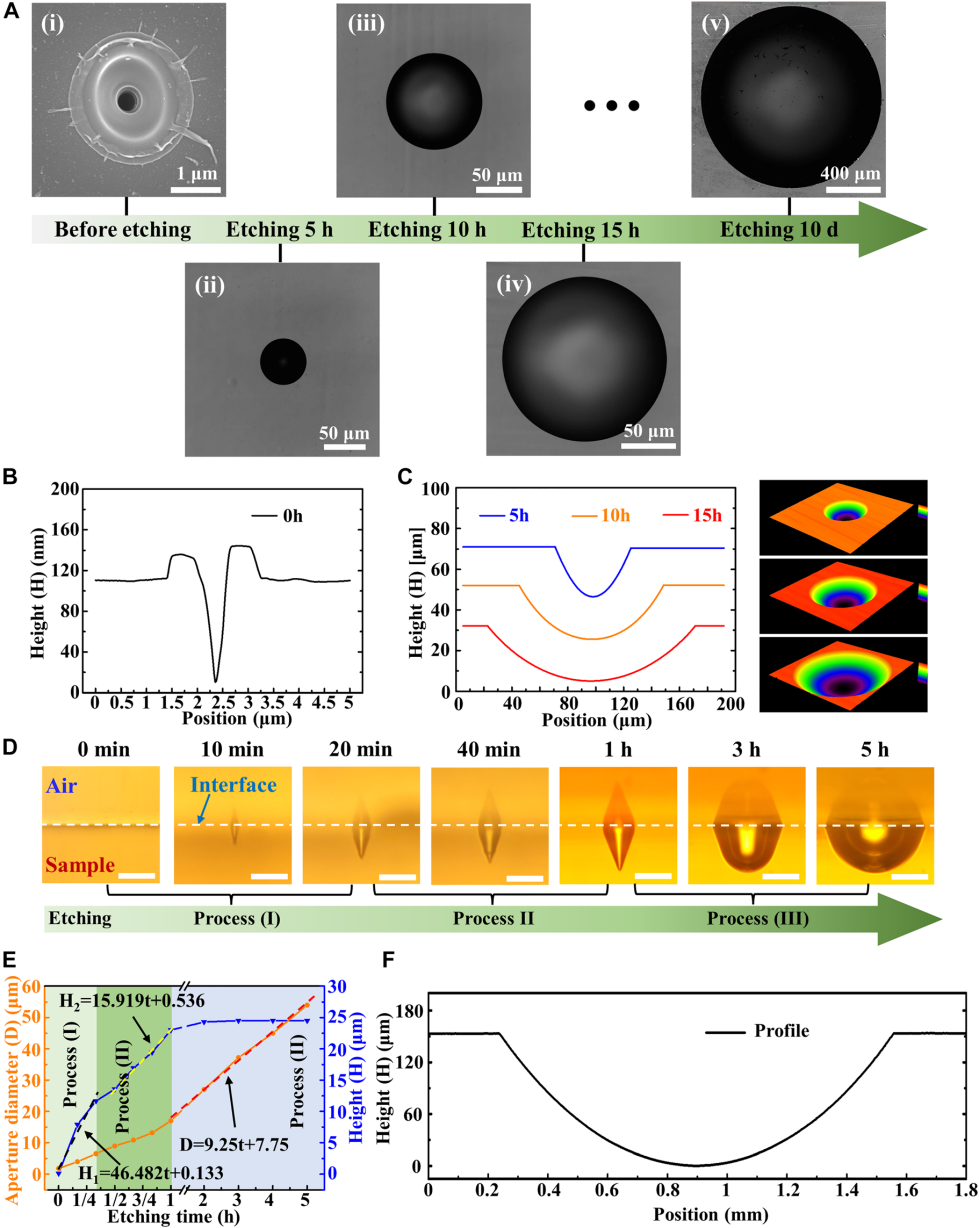
Process of microlens formation through BLB-assisted wet etching. (A) SEM image of the structure before etching (i), and LCSM images of the structure after etching for 5 h (ii), 10 h (iii), 15 h (iv), and 10 days (v), respectively. (B) Profile of the structure before etching measured by using atom force microscopy. (C) Profiles and 3D view of the structure after etching for 5 h, 10 h, and 15 h measured by using LCSM. (D) OTM images of structure morphology evolution with etching time from 0 min to 5 h. The scale bar is 20 μm. The dashed line represents the interface between the sample and the air. (E) Structural aperture diameters and heights that vary with etching time. The black dashed line and yellow dashed line represent the function of structural height increasing with etch time in Process I and Process II, respectively, and the red dashed line represents the function of structural aperture diameter increasing with etch time in Process III. (F) Profile of the structure after etching for 10 days measured by using LCSM.

The sample focused by a BLB with a BA of 2.34° was used as an example to demonstrate the process of microlens fabrication for further study, as shown in Figure 4A. According to Eqs. (1)–(4), the micro-Bessel region length of the beam was 422 μm. This value is 27 μm different from the micro-Bessel region length (395 μm) of the beam when BA is 2.5°. The morphology of the structure before etching was measured by using AFM, as shown in Figure 4B, which revealed that the structure was a tapered hole. The morphology of the etched microlens was measured by using LCSM. As shown in Figure 4C, The profile of the structure became smooth after etching for 5 h. The aperture diameters increased after etching for 10 h, and increased further after etching for 15 h. On the right is a 3D view of them. This trend shows that larger structures can be obtained by prolonging the etching time. After 10 days of etching, the size of the structure had even increased from the order of micrometers to the order of millimeters. Figure 4F shows the profile of the structure. The structure had an aperture diameter of 1.32 mm and can be used as a pupil lens in an Integral Field System [33].

Therefore, this technology not only allows the NA of the microlens to be adjusted by changing the BA in the Bessel phase but also allows the size of the microlens to be adjusted by changing the etching time. Figure 4D shows the structural morphology of etching time from 0 min to 5 h. It should be noted that the front and sides of the sample were polished. Since the sample was not placed absolutely horizontally when the structures were photographed, the OTM camera also captured side images of the samples. Therefore, the structures are shown in the images as symmetrical structures with the interface as the plane of symmetry. In each etching process, the diameter and height of the structure have different increasing trends. We show these trends in the form of data in Figure 4E. The modified region has a faster etching rate than the unmodified region. Therefore, within 20 min of etching time (Process I), the expanded region of the structure was mainly the modified region, and the axial size growth rate of the structure was much faster than the radial size growth rate of the structure. After the end of Process I, although the axial modified region had not been completely removed, the modified degree of the remaining modified region was weak. Next, the remaining weak modified region was removed during etching time from 20 min to 1 h (Process II), and the etching rate was between Process I and Process III, so Process II was called the transition between Process I and Process III. After etching for 1 h (Process III), the modified region had been completely removed. After that, the structure etched at the same rate in both axial and radial directions. The height was almost unchanged because the sample surface and bottom were etched at the same rate. In Figure 4E, we fitted the function of the structure height in Process I (the black dashed line) and Process II (the yellow dashed line) with the etching time, and also fitted the function of the structure aperture diameter in Process III (the red dashed line) with the etching time. The first one mainly reflects the etching law of the modified region. The middle one mainly reflects the etching law of the weak modified region between the modified region and the unmodified region. The last one mainly reflects the etching law of the unmodified region. And the three functions are as follows:

(5) $$H_{1}=46.482t+0.133$$

(6) $$H_{2}=15.919t+0.536$$

(7) D=9.25t+7.75

where *H*_1_ and *H*_2_ are the heights of the structure in Process I and Process II, respectively, *D* is the aperture diameter of the structure, and *t* is etching time. The slope in Eq. (5) indicates that the etching rate of the modified region is 46.482 μm h^−1^, the slope in Eq. (6) indicates that the etching rate of the weak modified region is 15.919 μm h^−1^, and half of the slope in Eq. (7) indicates that the etching rate of the unmodified region is 4.625 μm h^−1^. The etching rate of modified area was about 10 times that of unmodified area. Therefore, in Process I, the diameter and height of the structure increased rapidly. Since most of the modified region had been removed in Process I, the etching rate of the remaining weak modified region in Process II decreased, and the increase rate of the diameter and height of the structure was slowed down. In Process III, since the bottom of the structure and the surface of the sample were etched at the same rate, the height of the structure in the process remained basically unchanged, and the diameter of the structure increased steadily.

The above phenomena help to explain the change in the structure of the laser-induced fused silica and its interaction mechanism with HF. Fused silica is composed of SiO_2_, which is an amorphous structure in which a Si atom is surrounded by four O atoms connected by Si–O bonds. The structure of the fused silica is a random continuous network composed of numerous SiO_4_ tetrahedron-shaped crossing connections – the atomic structure inside the fused silica is in a state of long-range disorder. This structure consists of a network of crossing connections containing numerous closed loops surrounded by head-to-tail Si–O bonds, leading to the formation of numerous irregular X-ring structures, where X is the number of Si–O bonds in a closed loop, with a range of 3–9 [34, 35]. Schematic of the structure is shown in Figure S8. A six-ring structure possesses the maximum number and also the most stable structure. A series of nonlinear effects, such as multiphoton absorption and avalanche ionization, can be produced when a femtosecond laser pulse focuses on the surface of a material. When the laser energy is deposited into the material near the focus, the internal structure of the molten silicon changes greatly due to the extremely high photon density [36, 37]. When focused by a femtosecond laser, the relative number of three-ring and four-ring structures in the focused area increases, and the relative number of other types of ring structures decreases [38]. The angles of the Si–O–Si bonds bridged by three-ring and four-ring structures are decrease by a substantial amount [39], leading to the formation of compact and unstable structures in the focused region [40, 41]. The decrease in the angle of the bridging bond causes the valence electrons outside the O atom to occupy a deformed structure, and the deformed valence electrons in turn increase the reactivity of the O atom [42]. When silica interacts with HF, Si–O bonds break, the O atom combines with H^+^ in the HF solution, and the Si atom combines with F^−^ in the HF solution to form Si–OH and ≡SiF, respectively. The equation for the reaction between SiO_2_ and HF is as follows [34]:

(8) $$\equiv \text{Si}-\text{O}-\text{Si}\equiv +{\text{H}}^{+}{\text{F}}^{-}\to \text{Si}-\text{OH}+\text{SiF}$$

Because of the enhanced reactivity of the O atoms in the three-ring and four-ring structures, the structures are more likely to be broken by the HF solution through the reaction.

In the microscopic Raman spectrum of the fused silica displayed in Figure S8, two characteristic Raman peaks, P_1_ and P_2_, are observable at positions 495 and 606 cm^−1^, which represent the four-ring and three-ring structures in the internal structure network of the fused silica, respectively [26]. Peak P_1_ overlaps the wider principal spectral band at a wavenumber of 445 cm^−1^, increasing the difficulty of analysis and decreasing the accuracy of the data. Moreover, for the fused silica modified through femtosecond laser exposure, the main influence on the acid etching rate is the three-ring structure, exhibiting clearer changes in bond angle. Therefore, peak P_2_ was chosen as the target for research. We drew a spline curve (represented by the dashed line in Figure S8) below peak P_2_ as the baseline to analyze the area of the region surrounding peak P_2_ and the baseline. The area between peak P_2_ and the baseline in the modified area (red line) is slightly larger than the area between peak P_2_ and the baseline in the unmodified area (black line); hence, the relative number of three-ring structures in the modified area is larger than that in the unmodified area, which facilitates higher-efficiency chemical etching in the modified area.

### 3.3 The fabrication and imaging characterization of MLA with multiple NAs

**Figure 5:**
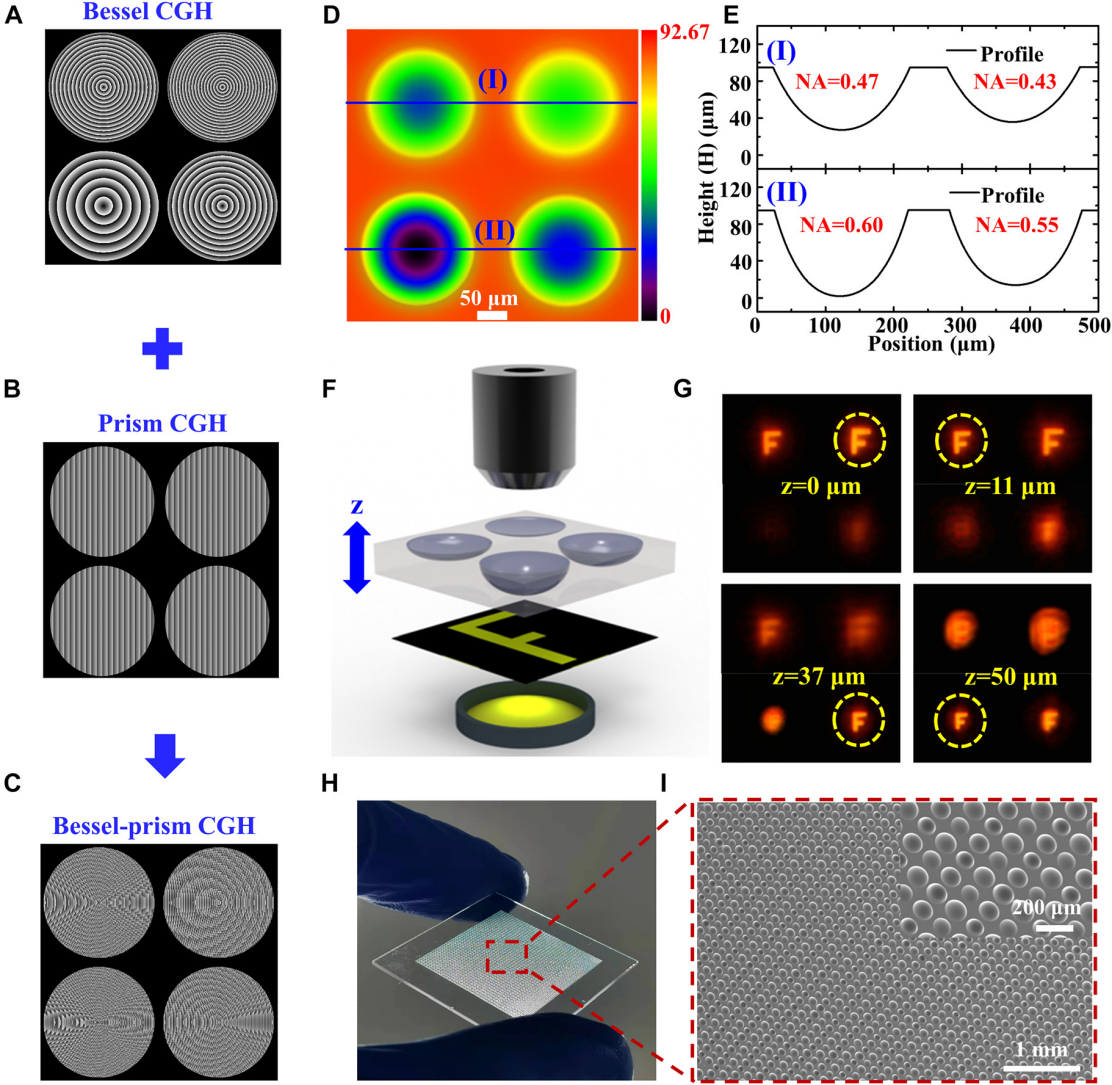
The designed Bessel-prism CGH was loaded into SLM to fabricate the MLA with multiple NAs, and the multi-plane imaging performance of the MLA was characterized. (A) CGH with a variety of Bessel phases. (B) Prism CGH was used to deflect BLBs and separate them from the zero-order beam. (C) The Bessel-prism CGH produced by combining the Bessel phase in (A) and the prism phase in (B). (D) A top view of the array composed of four microlenses with the same aperture diameter and different NAs was obtained using LCSM. (E) Corresponding cross-section profiles of microlenses in (D). (F) Schematic of the imaging system for the MLA. (G) Image display of MLA at different positions in *z* direction. The dashed yellow circles mark the clearest image at each position. (H) Large-area MLA photograph and (I) SEM image fabricated in fused silica. The inset is the magnified SEM image.

Finally, we fabricated an MLA with different NAs by using single pulse BLBs. Figure 5A displays the Bessel CGH, in which the BAs of the four phases were 2.30°, 2.24°, 2.19°, and 2.09°. The lengths of the corresponding micro-Bessel regions were 430 μm, 441 μm, 451 μm, and 473 μm, respectively. And the difference between them and the micro-Bessel region length at 2.5° BA was 35 μm, 46 μm, 56 μm and 78 μm, respectively. To remove the interference from the zero-order light on the fabrication process, we combined the Bessel CGH with the 70° prism CGH, as shown in Figure 5B, to obtain the Bessel-prism CGH, as shown in Figure 5C. The Bessel-prism CGH was loaded into the SLM, and the Gaussian beam modulated by the SLM was converted into four micro-BLBs through the telescope system, which were focused on the surface of the fused silica sample. After 20 h of etching with an HF solution, the MLA in Figure 5D was generated, and Figure 5E displays its profile. The aperture diameters of the four microlenses in the MLA were highly similar, but the NAs were different. The NA of each microlens is marked above its respective profile and is 0.43, 0.47, 0.55, and 0.60.

The imaging capability of the MLA was demonstrated by using the imaging system in Figure 5F, which consists of a source of white light, a mask “F”, the MLA and a microscope objective. The MLA can move up and down in the *z* direction. Mask “F” was placed between the white light source and the MLA, and the image obtained through the MLA was received by the microscope objective lens above it. By moving the MLA in the *z* direction to change the distance between it and the objective lens, the MLA exhibited multiple-plane imaging properties, as shown in Figure 5G. When the imaging of the microlens with the smallest NA in the MLA was the clearest, the current *z*-value was recorded as 0 μm. Then, continued to move. The *z*-values of the other three microlenses in the MLA when they were the clearest were 0 μm, 11 μm, 37 μm and 50 μm, respectively. The dynamic process is shown in Movie S1. Intensity Point Spread Function (IPSF) was used to evaluate the focusing ability of microlens. The IPSF test system was constructed by using the method described in reference [43]. The system consists of a supercontinuum laser source (SuperK EXTREME, NKT Photonics, Denmark) (*λ* = 650 nm), a beam expander (Thorlabs, GBE03-B), a microscopic objective lens (100×/NA = 0.9) and a charge-coupled device (Lm11059C, Lumenera, Canada). We characterized the IPSF of MLA fabricated by the proposed technology, and demonstrated the microlens with NA = 0.43 as an example (Figure S9A), which indicates that the microlens fabricated by the proposed technology has a good focusing ability [44]. And the modulation transfer function (MTFs) was calculated from IPSF to quantify the imaging resolution ability of the microlens [45]. By an empirical criterion of 0.2, the microlens fabricated with the proposed technology shows high spatial resolution (Figure S9B) [44, 45]. Therefore, the MLA can perform multiple-plane imaging, allowing it to potentially be used in applications such as 3D imaging, optical communication, integrated optics, and cell detection.

**Video 1:** 

In addition, combined with the flying punch method, large area MLAs can be fabricated efficiently [46]. The proposed technology is a parallel fabricating technology, since a single exposure can obtain an array of modified points in the femtosecond laser modification procedure. Therefore, the fabricating efficiency of this technology is *N* times that of the traditional flying punch technology, where *N* is the number of modification points per exposure. Figure 5H displays the fabricated large area MLA. The MLA size was 2 × 2 cm, and there were 6400 microlenses in total. The average fabricating time of per microlens in the array was about 11 s when the etching time was 20 h.

## 4 Conclusions

In summary, a technology for fabricating an MLA is proposed to generate BLBS with multiple BAs by modulating a Gaussian femtosecond laser. Modified regions with different volumes were generated by using a single pulse on the fused silica sample, and the MLA with different NAs was formed after HF etching, which can achieve multiple-plane imaging. The NA of the microlens fabricated through this technology can be adjusted between 0.06 and 0.65, which is the maximum range to have ever been reported. The maximum change in NA can reach 0.45–0.65 with the same aperture diameter (within error range of ±2 μm) in a certain range. Furthermore, by prolonging the etching time, the size of the microlens can be increased from the order of micrometers to the order of millimeters scale, providing a new concept for the fabrication of lens. The technology allows parallel fabrication of MLAs by changing the pattern determined by the CGH, and it is *N* (the number of modification points per exposure) times more efficient than traditional technology. This technology has potential applications in 3D imaging, optical communication, integrated optics, and cell detection.

## Supplementary Material

Supplementary Material Details

Supplementary Material Details
