# Supplementary material for: Morphology adjustable microlens array fabricated by single spatially modulated femtosecond pulse
Source: Nanophotonics. 2022 Jan 4;11(3):571–81. doi: 10.1515/nanoph-2021-0629 (PMC11501908; doi:10.1515/nanoph-2021-0629)
Supplement: Supplementary file 3 — Supplementary Material Details [file j_nanoph-2021-0629_suppl_001.docx]

Supplement Materials

Morphology Adjustable Microlens Array Fabricated by Single Spatially Modulated Femtosecond Pulse

Yang Liu^1^, Xiaowei Li^*1,2^, Zhipeng Wang^1^, Bin Qin^1^, Shipeng Zhou^1^, Ji Huang^3^ and Zhulin Yao^4^

*^1^ Laser Micro/Nano Fabrication Laboratory, School of Mechanical Engineering, Beijing Institute of Technology, Beijing 100081, China*

*^2^* *Beijing Institute of Technology Chongqing Innovation Center, Chongqing 401120, PR China.*

*^3^ National Institute of Metrology, Beijing 100029, China*

*^4^ AECC Shenyang Engine Research Institute, Shenyang 110066, China*

* E-mail: lixiaowei@bit.edu.cn


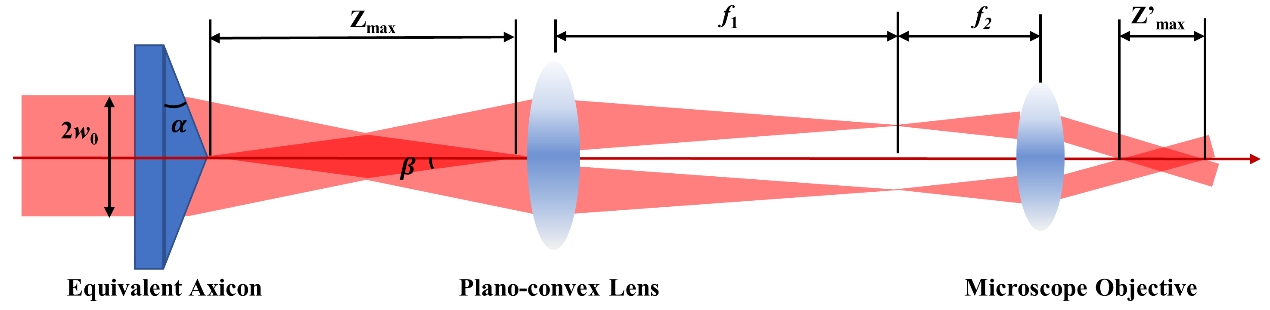


**Figure S1** **Schematic of Bessel beam generation and demagnification.** The equivalent axicon lens is used to generate BLB, and the telescopic system composed of plano-convex lens and microscopic objective lens is used to demagnify BLB. Where ***β*** is the exit angle, ***α*** is the BA, ***n*** is the refractive index of the axicon, which is 1.45, ******and ******are the length of the Bessel region before and after it passes through the telescopic system, respectively, ***w_0_*** is the beam radius at the waist of the incident Gaussian beam, ***f*_1_** and ***f_2_*** are the focal length of the plano-convex lens and the equivalent focal length of the microscope objective lens, respectively.


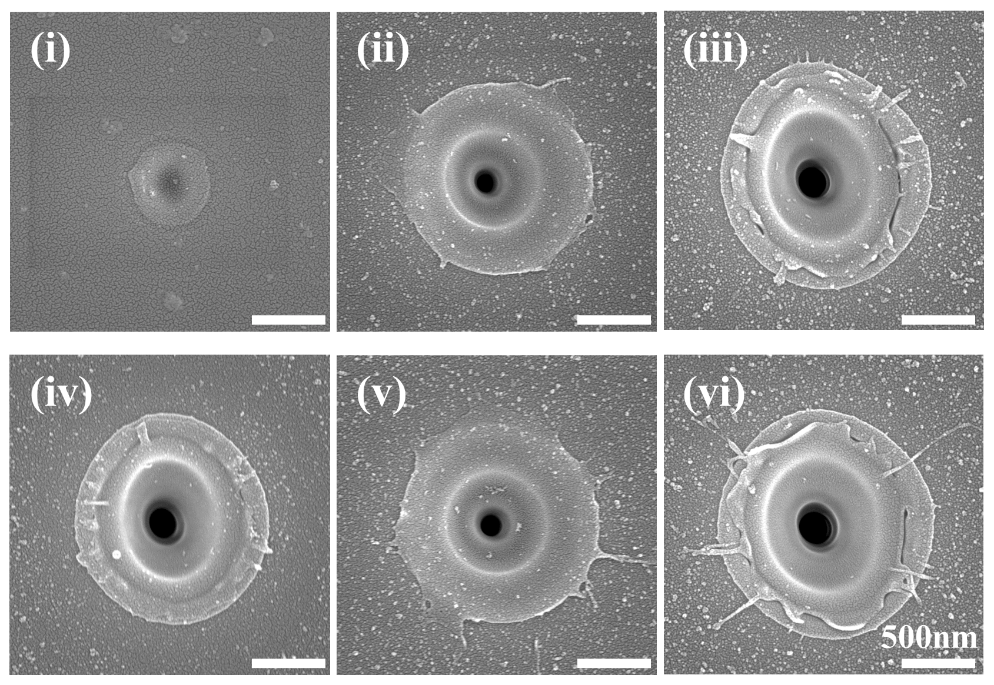


**Figure S2** **SEM images of modified regions defined by the six BLBs.** The BA in these BLBs is (i) 2.5°, (ii) 2.4°, (iii) 2.3°, (iv) 2.2°, (v) 2.1° and (vi) 2.0°, respectively. The scale is 500 nm.


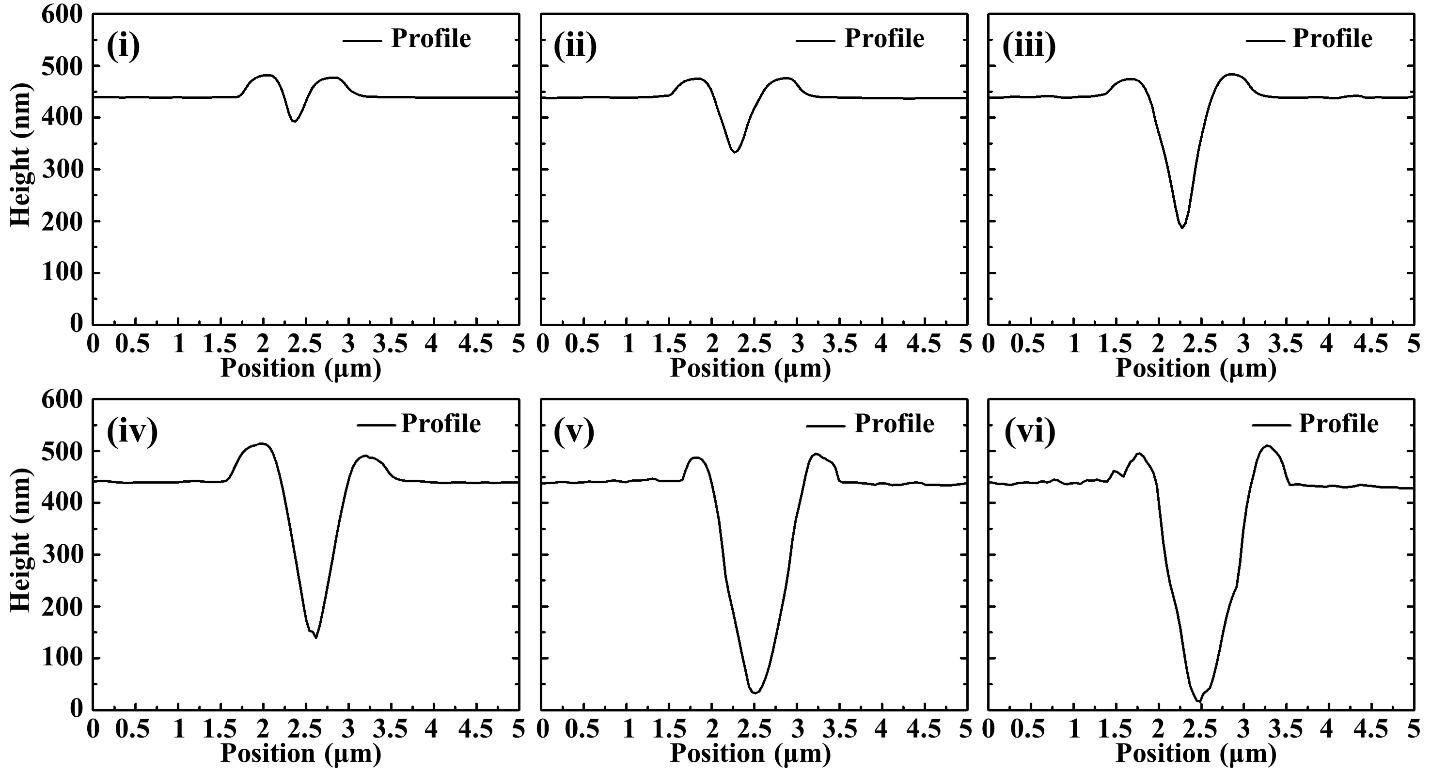


**Figure S3** **Profiles of modified regions defined by the six BLBs measured by AFM.** The BA in these BLBs is (i) 2.5°, (ii) 2.4°, (iii) 2.3°, (iv) 2.2°, (v) 2.1° and (vi) 2.0°, respectively.

**Calculation equations of focal length and NA of microlens:**

$R=\frac{h^{2}+r^{2}}{2h}$ (S1)

$f=\left|\frac{R\sin\theta}{\sin\left[\left(n-n_{0}\right)\theta\right]}\right|$ (S2)

$NA=\frac{r}{f}$ (S3)


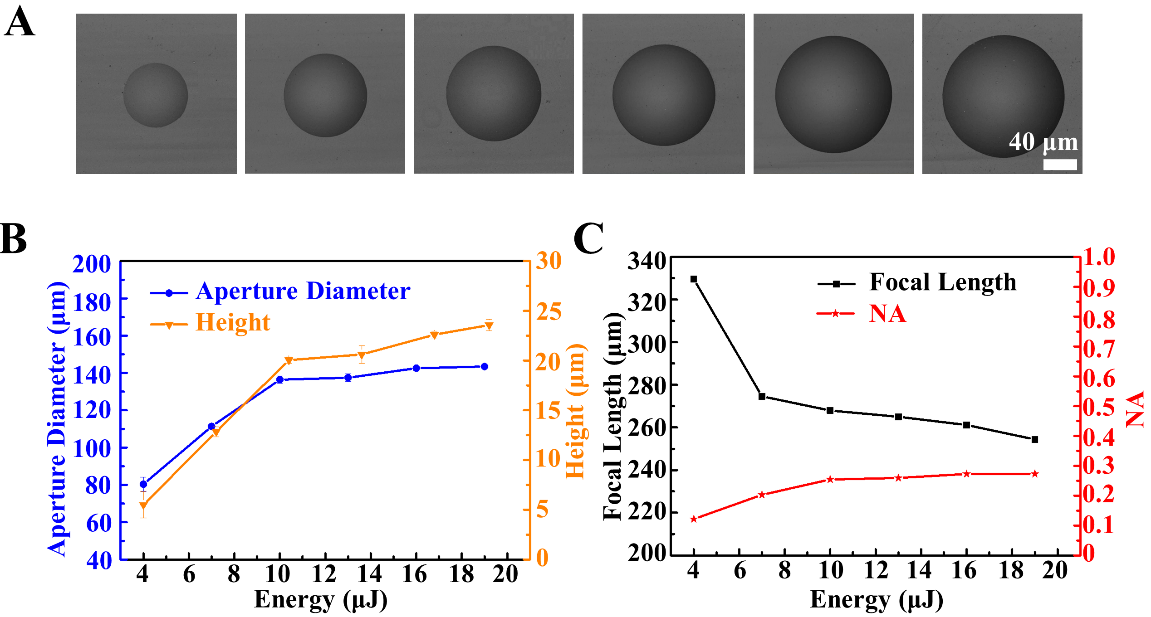


**Figure S4** **Microlenses yielded by irradiation of Gaussian lasers with a variety of pulse energies.** (A) LCSM images of the microlenses after etching. (B) Relationship among apertures, heights, and pulse energies. (C) Calculated focal length and NA corresponding to (B).


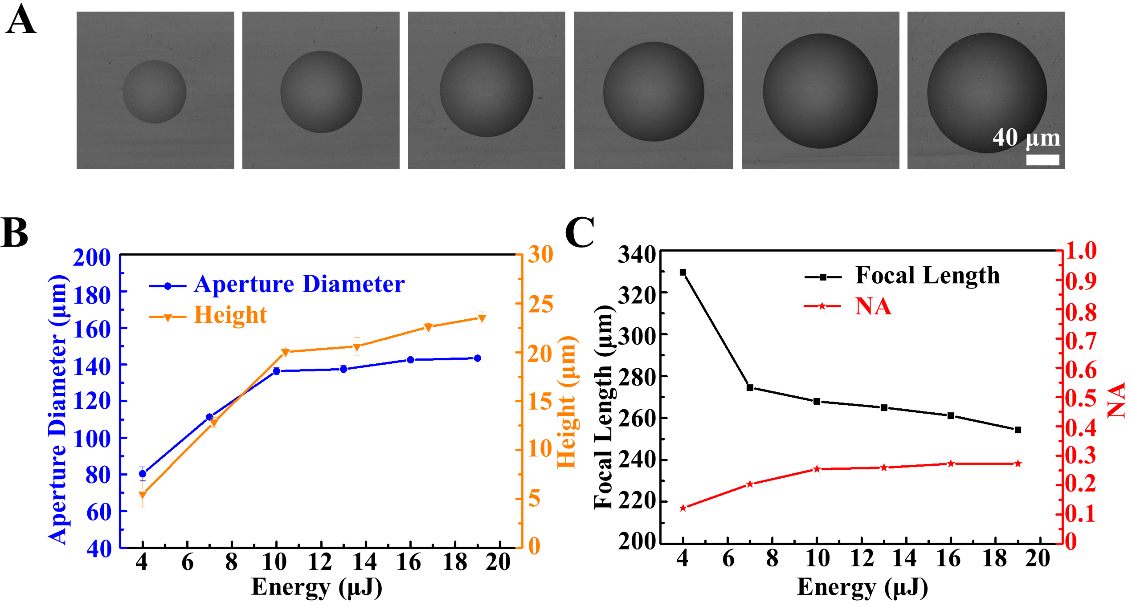


**Figure S5** **Microlenses yielded by irradiation of Gaussian lasers with a variety of pulse numbers.** (a) LCSM images of the microlenses after etching. (b) Relationship among apertures, heights, and pulse numbers. (c) Calculated focal length and NA corresponding to (b).


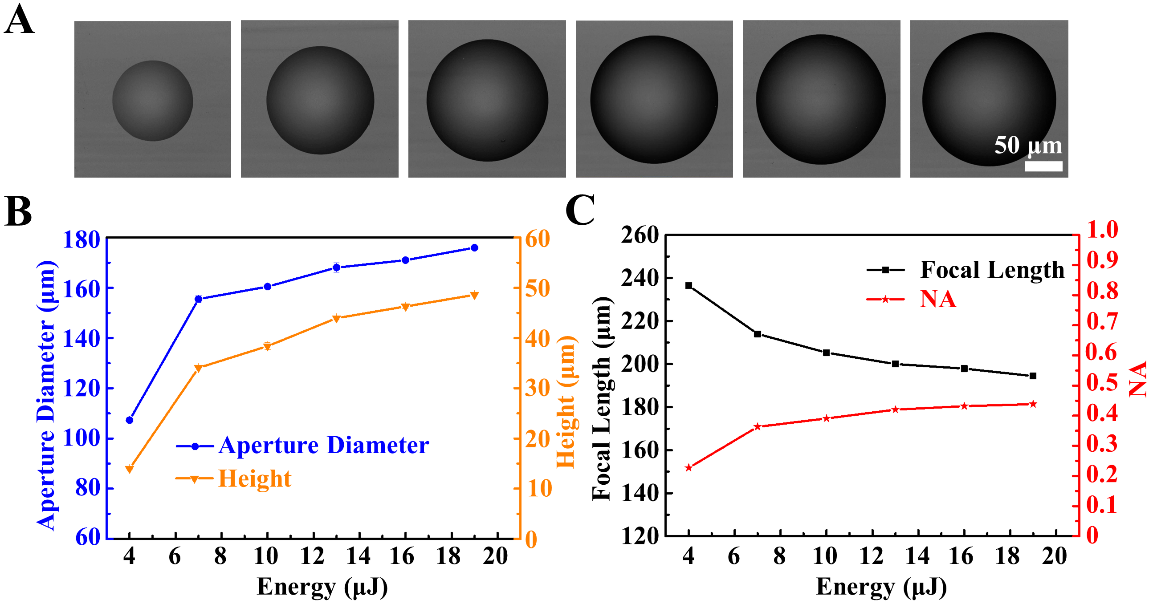


**Figure S6** **Microlenses yielded by irradiation of BLBs with a variety of pulse energies.** (A) LCSM images of the microlenses after etching. (B) Relationship among aperture diameters, heights, and laser energies. (C) Calculated focal length and NA corresponding to (B).


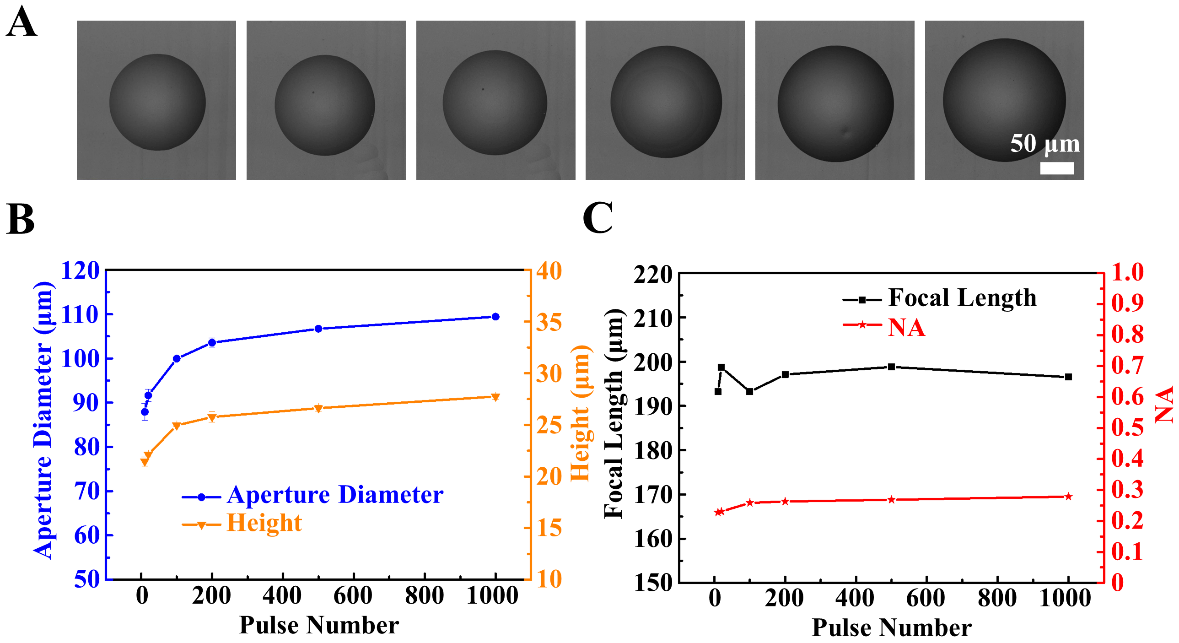


**Figure S7** **Microlenses yielded by irradiation of BLBs with a variety of pulse numbers.** (A) LCSM images of the microlenses after etching. (B) Relationship among aperture diameters, heights, and pulse numbers. (C) Calculated focal length and NA corresponding to (B).


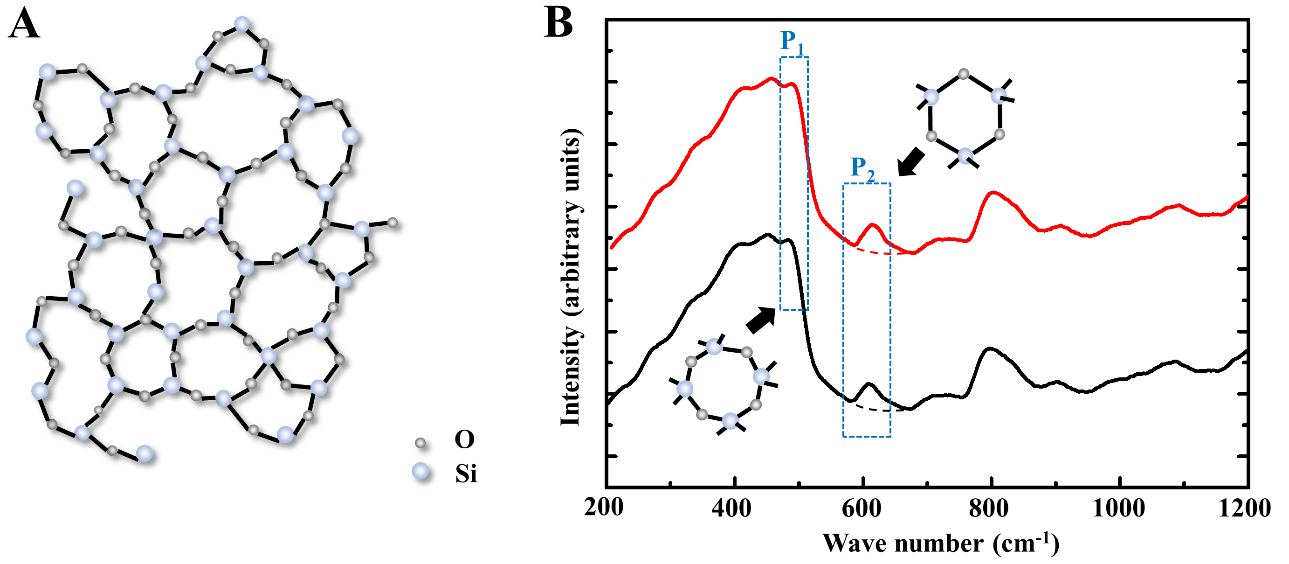


**Figure S8** **Schematic of the internal structure network of the fused silica and the results of Raman measurements of modified and unmodified regions irradiated by BLB.** (A) Two-dimensional diagram of amorphous structure in fused silica. (B) Results of Raman measurements of modified (red line) and unmodified (black line) regions.


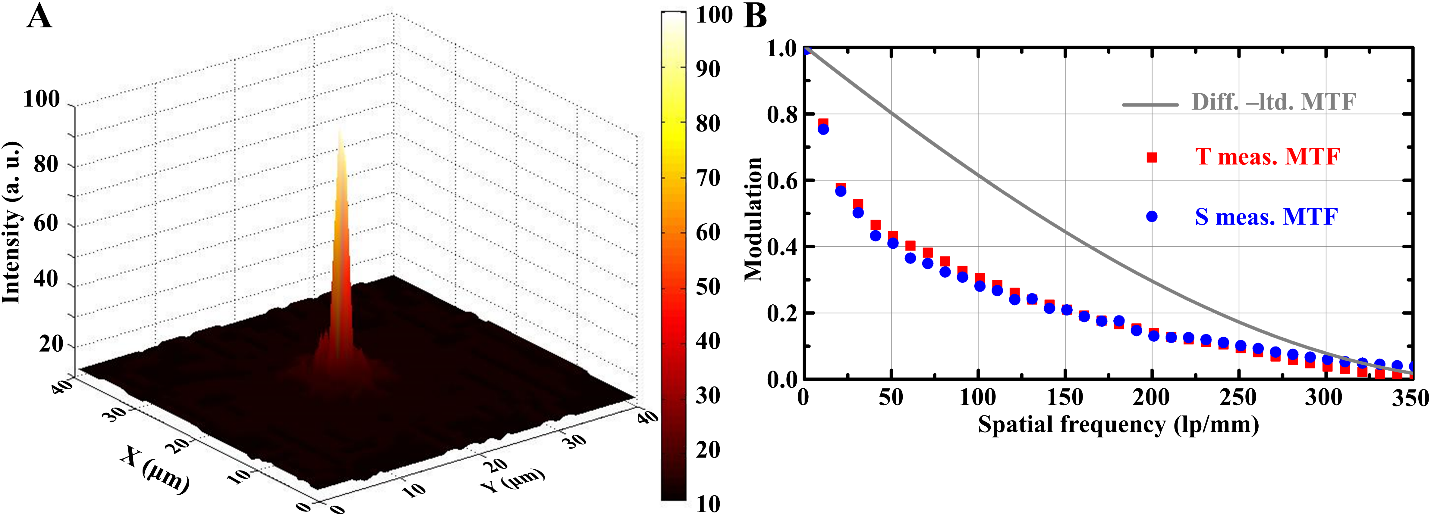


**Figure S9** (A) The measured IPSF of one microlens in the MLA, and (B) the MTF calculated from the IPSF.

**Table S1** **Comparison of the maximum NA of the microlens processed by the technology mentioned in this article and the maximum NA of the microlens processed by the femtosecond laser technology in other reports.**

| Materials | Method | Maximum NA | Reference |
| --- | --- | --- | --- |
| Silica | FLAE | 0.65 | This work |
| Silica | FLAE | 0.4 | [1] |
| Silica | FLAE | 0.26 | [2] |
| Silicon | FLAE | 0.137 | [3] |
| PDMS | Femtosecond  laser scanning | 0.17 | [4] |
| Photoresist | Femtosecond  laser scanning | 0.46 | [5] |
| Polyimide | Laser ablation | 0.5 | [6] |
| PMMA | Laser-Induced structural modification | 0.069 | [7] |

**References**

[1] Fan H, Cao XW, Wang L, et al. Control of diameter and numerical aperture of microlens by a single ultra-short laser pulse. Opt. Lett. 2019, 44(21), 5149-5152.

[2] Chen F, Liu HW, Yang Q, et al. Maskless fabrication of concave microlens arrays on silica glasses by a femtosecond-laser-enhanced local wet etching method. Opt. Express 2010, 18(19), 20334-20343.

[3] Deng ZF, Yang Q, Chen F, et al. Fabrication of large-area concave microlens array on silicon by femtosecond laser micromachining. Opt. Lett. 2015, 40(9), 1928-1931.

[4] Yong JL, Chen F, Yang Q, et al. Rapid fabrication of large-area concave microlens arrays on PDMS by a femtosecond laser. ACS Appl. Mater. Inter. 2013, 5(19), 9382-9385.

[5] Wu D, Wu SZ, Niu LG, et al. High numerical aperture microlens arrays of close packing. Appl. Phys. Lett. 2010, 97(3), 031109.

[6] Zimmer K, Hirsch D, Bigl F. Excimer laser machining for the fabrication of analogous microstructures. Appl. Surf. Sci. 1996, 96, 425-429.

[7] Ou Y, Yang Q, Chen F, et al. Direct fabrication of microlens arrays on PMMA with laser-induced structural modification. IEEE Photonic. Tech. L. 2015, 27(21), 2253-2256.
